# Meningioma of Foramen Magnum Causing Drop Attacks

**DOI:** 10.1155/2015/214563

**Published:** 2015-02-22

**Authors:** Amit Mahore, Raghvendra Ramdasi, Sandip Mavani, Vithal Rangarajan, Manoj Patil, Prashant Sathe, Juhi Kawale, Vishakha Tikeykar

**Affiliations:** ^1^Department of Neurosurgery, Surana Hospital & Research Centre, Malad, Mumbai 400064, India; ^2^Department of Neurosurgery, King Edward Memorial Hospital and Seth Gordhandas Sunderdas Medical College, Parel, Mumbai 400012, India; ^3^Department of Medicine, King Edward Memorial Hospital and Seth Gordhandas Sunderdas Medical College, Parel, Mumbai 400012, India; ^4^Department of Pathology, Bombay Hospital Institute of Medical Sciences & Research, New Marine Lines, Mumbai 400020, India

## Abstract

A 52-year-old female presented with frequent episodes of falls without loss of consciousness. These episodes lasted for brief period followed by full neurological recovery. Magnetic resonance imaging (MRI) of the brain showed foramen magnum meningioma encasing left vertebral artery. The patient had dramatic improvement after excision of the tumor.

## 1. Introduction

Meningioma is the commonest tumor in the region of foramen magnum [1]. It often presents with occipitocervical pain, long tract signs, and lower cranial nerve deficits [2, 3]. Drop attack as a presenting symptom of meningioma of foramen magnum has never been reported. We report an adult patient with this unique presentation and discuss the differential diagnosis, diagnostic approach, and a brief review of literature.

## 2. Case Report

A 52-year-old female, a known case of hypertension, presented with sudden episodes of fall without loss of consciousness for the last 5 months. Each episode used to last for approximately 1 minute followed by full neurological recovery. The frequency of such drop attacks was 1-2 per day. It was associated with giddiness. Neurological examination was normal apart from mild hyperreflexia in all four limbs.

**Figure 1:**
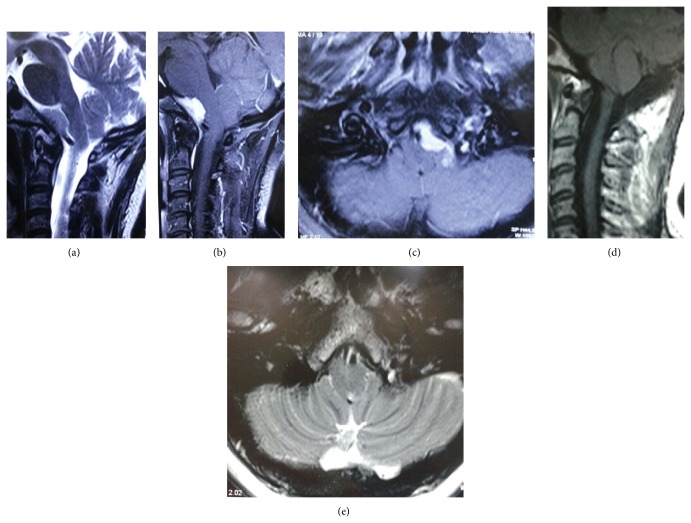
(a) T2 weighted sagittal image; (b) postcontrast T1 weighted sagittal image; (c) postcontrast T1 weighted axial image: magnetic resonance imaging (MRI) showing anterolaterally placed, homogenously enhancing dural based lesion in the foramen magnum encasing the left vertebral artery suggestive of meningioma. (d) T1 weighted sagittal image and (e) T1 weighted axial image: postoperative MRI showing complete excision of tumor.

Twenty-four-hour electroencephalography (EEG) was done by a neurologist which was normal. Electromyography (EMG) and nerve conduction velocity (NCV) of all four limbs were also normal. Cardiovascular workup which included echocardiograms (ECG) and Holter monitoring were normal. Magnetic resonance imaging (MRI) revealed anterolaterally placed, homogenously enhancing dural based lesion in the foramen magnum encasing the left vertebral artery suggestive of meningioma (Figures 1(a), 1(b), and 1(c)).

Patient underwent surgery by posterior approach. Total excision of the tumor followed by augmentation duraplasty was performed via midline suboccipital craniectomy and removal of C1 arch. Tumor was arising from anterolateral dura of foramen magnum, firm in consistency, vascular, and nonsuckable. It was excised in piecemeal, baring the left vertebral artery and left posterior inferior cerebellar arteries (PICA). Complete excision was done with coagulation of dural base.

Patient had a dramatic recovery from her symptoms postoperatively. At follow-up of 18 months after the surgery patient is disease-free (Figures 1(d) and 1(e)).

**Figure 2:**
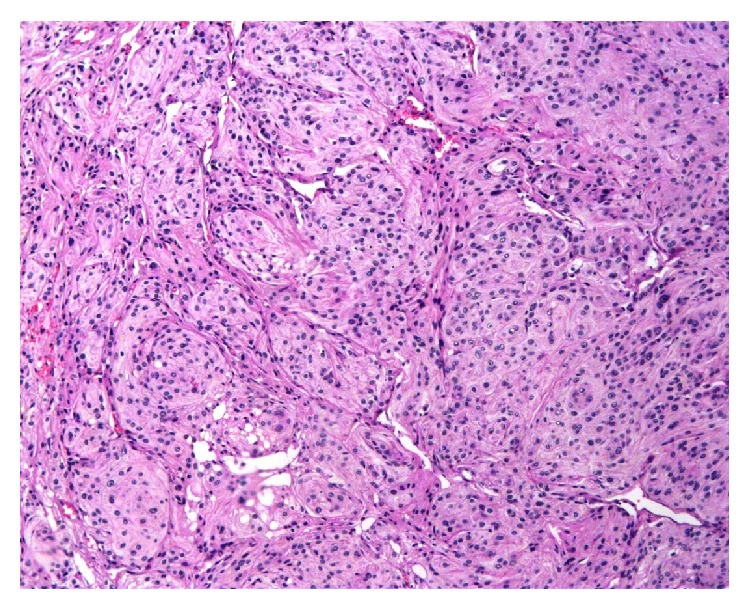
Photomicrograph (H& E, 10x) showing lobular arrangement of meningothelial cells with syncytial distribution at the periphery suggestive of meningothelial meningioma.

Histopathological examination showed the lesion to be meningothelial meningioma (Figure 2).

## 3. Discussion

**Figure 3:**
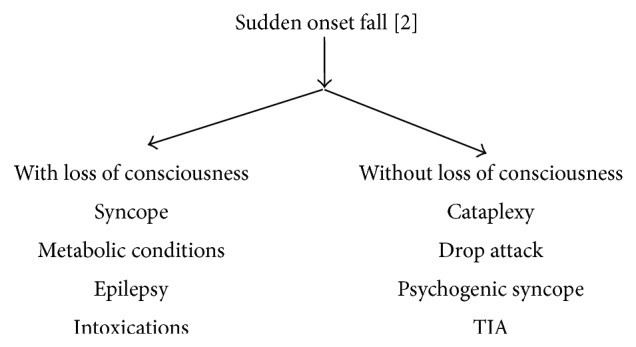
Causes of sudden onset fall.

Fall of sudden onset can be associated with or without loss of consciousness. Falls with loss of consciousness can be due to syncope or nonsyncopal conditions like metabolic disorders including hypoglycaemia: hypoxia, epilepsy, and intoxications. Falls without loss of consciousness can be due to cataplexy, drop attacks, psychogenic “syncope” (somatization disorders), and transient ischaemic attacks (Figure 3).

Syncope is defined as a transient, self-limited loss of consciousness, usually leading to fall. The onset of syncope is relatively rapid, and the subsequent recovery is spontaneous, complete, and usually prompt. The underlying mechanism is a transient global cerebral hypoperfusion. The causes of syncope include neurally mediated reflex syncopal syndromes, cardiac arrhythmias, orthostatic hypotension, and cerebrovascular conditions [8].

Drop attacks are sudden falls without loss of consciousness that are not precipitated by a specific stimulus, occur with abrupt onset and without warning, and are followed by a rapid return to baseline. A range of localizations for drop attacks is possible, but most commonly lower brainstem or spinal cord structures are implicated. Drop attacks generally indicate transient impairment of bilateral central nervous system structures involved in maintenance of postural muscle tone and balance [9]. Tumarkin otolithic catastrophes (or crises) are drop attacks without associated autonomic or neurologic symptoms in patients with severe vestibular disease, usually due to Ménière disease [10]. The causes of drop attacks include cervical cord compression, vertebrobasilar ischemia [11], inner ear disorders (e.g., Ménière disease and migrainous vertigo) [10], hydrocephalus, and myxedema [12]. We found four cases of craniospinal tumors causing drop attacks (Table 1). That includes third ventricular meningioma, fourth ventricular arachnoid cyst, and choroid plexus papilloma [4, 5, 7]. Drop attacks were attributed to the hydrocephalus caused by the lesions. One case of C2 neurinoma causing drop attacks has been reported presumably due to vertebral insufficiency [6]. Our case is fifth case of the tumor causing drop attacks and first in the region of foramen magnum. Bow Hunter's syndrome is symptomatic vertebrobasilar insufficiency due to mechanical stenosis or occlusion of a vertebral artery (VA) at C1-C2 during rotation of the head. The slow growth of tumor gives enough time for collateral pathways to develop compensating for reduced blood flow even due to dominant VA compression. Hence symptoms due to tumoral compression of vertebral arteries are rare [13]. Angiographic studies were not done to see the dominant VA in our patient due to the economic constraints. The drop attacks in the present patient could have been due to the transient vertebrobasilar insufficiency and medullary compression related to head movement as vertebral artery was encased by the tumor. Transient obstruction of flow of cerebrospinal fluid (CSF) leading to the development of an increase in intracranial pressure can also lead to drop attacks.

In elderly patients with sudden falls and presumed drop attacks, the absence of a history of loss of consciousness is unreliable. More than two-thirds of such patients are in fact found to have forms of syncope, epilepsy, and cerebrovascular disorders like vertebrobasilar insufficiency. Drop attacks due to vertebrobasilar insufficiency are commonly accompanied by other event-related neurologic manifestations (e.g., visual loss, diplopia, vertigo, and numbness) in addition to the sudden loss of postural tone in the legs [14]. Therefore ECG, EEG and relevant investigations should be done in the patients presenting with episodic falls.

Foramen magnum (FM) is bounded anteriorly by lower third of the clivus and upper edge of the body of C2, laterally by jugular tubercles and upper aspect of C2 laminas, posteriorly by anterior edge of the squamous occipital bone and C2 spinous process.

Meningiomas of Foramen magnum represent around 3% of all meningiomas and 1% of all primary brain tumors. These represent 70% of all tumors in that region. The lesion is often large at diagnosis because of their slow-growing rate, long interval since the first symptom, and the wide subarachnoid space at this level [1, 15]. These present with occipitocervical pain, long tract signs, and lower cranial nerve deficits [2, 3]. Meningiomas in the foramen magnum frequently elude early diagnosis because their ill-defined symptoms mimic cervical spondylosis, multiple sclerosis, syringomyelia, normal pressure hydrocephalus, amyotrophic lateral sclerosis, Chiari I malformation, carpal tunnel syndrome, and intramedullary or extramedullary tumors [16]. Meningioma of the foramen magnum when unrecognised may lead to progressive myelopathy with quadriplegia, dysphagia, and sphincter disturbance [17].

## 4. Conclusion

Foramen magnum meningioma may rarely present with drop attacks and should be considered in the differential diagnosis of the conditions causing drop attacks. Magnetic resonance imaging (MRI) may clinch the organic causes of drop attacks and help in early diagnosis of foramen magnum meningioma. It should be included in the diagnostic workup of patients presenting with drop attacks.

## Figures and Tables

**Table 1:** Tumors causing drop attack.

Author/year	Number	Age/sex	Site and nature of the tumor	Mechanism
Criscuolo and Symon (1986) [4]	1	—	3rd ventricular meningioma	Hydrocephalus
Lee et al. (1994) [5]	1	—	Posterior fossa arachnoid cyst	—
George and Laurian (1989) [6]	1	31/M	C2 neurinoma	Vertebral insufficiency
Pollack et al. (1995) [7]	1	2/Mch	Choroid plexus papilloma third ventricle	Hydrocephalus
Present case	1	46/F	Foramen magnum meningioma	Vertebral insufficiency and medullary compression

M: male, F: female, and Mch: male child.
